# Probing tribological evolution in atomically thin MoS_2_ at different scales

**DOI:** 10.3762/bjnano.17.40

**Published:** 2026-05-06

**Authors:** Xingzhong Zeng, Miao Zhang

**Affiliations:** 1 School of Intelligent Manufacturing, Hunan First Normal University, Changsha 410205, China; 2 Changsha Denghua Off-Campus Custodial Service Co., Ltd., Changsha, China

**Keywords:** atomic force microscopy, MoS_2_, strengthening effect, sub-nanoscale stick–slip motion

## Abstract

Atomic-scale stick–slip motion and the nanoscale strengthening effect are foundational phenomena in the nanotribology of atomically thin two-dimensional (2D) materials, yet their sub-nanoscale origins and load-dependent evolution remain insufficiently characterized. Herein, we systematically investigate the sub-nanoscale friction behaviors of atomically thin molybdenum disulfide (MoS_2_) under controlled loads using a calibrated atomic force microscope (AFM), with a focus on quantifying the strengthening effect and sub-nanoscale stick–slip motion. Our results reveal that the nanoscale strengthening effect intensifies with increasing applied load but weakens as the number of MoS_2_ layers increases, attributed to the reduced out-of-plane flexibility in thicker films. Critically, we identify the slip distance during the slip phase as a reliable metric for sub-nanoscale stick–slip motion. The slip distance increases with the frequency of sub-nanoscale stick–slip events and exhibits a load-dependent transition, remaining nearly constant under low loads before increasing with higher loads, and ultimately decreasing at ultrahigh loads. This transition arises from two competing mechanisms: under low loads, the evolution of interfacial contact quality dominates the strengthening effect and suppresses sub-nanoscale stick–slip motion. Under moderate-to-high loads, the puckering effect becomes dominant, enhancing sub-nanoscale stick–slip events and increasing slip distance. At ultrahigh loads, the nanoscale strengthening effect transitions to static friction, which quenches sub-nanoscale stick–slip motion and reduces slip distance. Furthermore, the load-dependent sub-nanoscale stick–slip motion is closely correlated with changes in tip–MoS_2_ contact area and contact geometry, both modulated by load and MoS_2_ layer thickness. These findings advance 2D material tribology from the nanoscale to sub-nanoscale, providing critical insights for designing low-friction coatings and high-performance micro/nanoelectromechanical systems (MEMS/NEMS).

## Introduction

Nanoscale friction is a pivotal factor limiting the performance and reliability of nanotechnology-enabled devices, including magnetic storage systems and micro/nanoelectromechanical systems (MEMS/NEMS) [1–2]. Unlike macroscale friction, nanoscale friction of two-dimensional (2D) materials exhibits unique size-dependent and interface-sensitive characteristics, such as contact area dependence [3], superlubricity [4–6], negative friction coefficients [7], and friction anisotropy [8]. A hallmark of nanoscale friction is atomic-scale stick–slip motion, first observed by sliding a tungsten tip over graphite, where the tip “sticks” to an atomic lattice site until the lateral force overcomes interfacial interactions, followed by an instantaneous “slip” to the next stable site [9]. This stick–slip motion is widely regarded as the elementary mechanism of energy dissipation in nanoscale friction, underpinning efforts to understand energy conversion at the atomic level.

However, recent advances have challenged this simplification: Maier et al. identified intermediate states during slip, suggesting slip is a complex, non-instantaneous process [10]. Lee et al. further revealed that nanoscale stick–slip motion consists of sub-nanoscale stick–slip events driven by in-plane tip apex motion and modulated by contact geometry via molecular dynamics (MD) simulations and frictional force microscopy (FFM) [11–12]. Despite these theoretical and simulation insights, experimental observation and quantification of sub-nanoscale stick–slip motion in 2D materials remain elusive, primarily due to the need for high resolution and precise control of tip–sample interactions.

Atomically thin 2D materials are ideal platforms for studying nanoscale and sub-nanoscale friction, owing to their atomic smoothness, well-defined crystal structure, and chemical inertness [13–15]. Molybdenum disulfide (MoS_2_) as a representative 2D materials is particularly promising because of its trilayered structure (one Mo layer sandwiched between two S layers) and tunable interlayer interactions, making it a model system for investigating the nanoscale and sub-nanoscale friction properties [5,16]. Previous studies on MoS_2_ have reported that nanoscale friction decreases with increasing layer thickness, and a transient nanoscale strengthening effect (enhanced friction in the initial sliding cycles) is attributed to either interfacial contact quality evolution or the puckering effect [17–18]. However, these studies focused on nanoscale phenomena; the sub-nanoscale origins of stick–slip motion remains unaddressed.

Here, we use a calibrated AFM to quantitatively study the sub-nanoscale friction behaviors of a single layer (1L) and four layers (4L) of MoS_2_ under different loads. We experimentally determined the sub-nanoscale stick–slip motion and quantified its dependence on load and layer thickness. The corresponding mechanisms were proposed to explain the friction behaviors. This work bridges the gap between MD simulations and experiments, advancing 2D material tribology toward the sub-nanoscale.

## Experimental

### Sample preparation

Single-layer and few-layer MoS_2_ were obtained from bulk MoS_2_ by mechanical exfoliation [19]. N-Doped Si covered with dry oxidation-generated 300 nm thick SiO_2_ was used as the substrate. Prior to exfoliation, substrates were cleaned sequentially in acetone, ethanol, and deionized water via ultrasonic cleaning and then dried with high-purity nitrogen.

### Sample characterization

MoS_2_ layer thickness was determined via AFM (MFP-3D, Asylum Research) and Raman spectroscopy (inVia Reflex, Renishaw). AFM topographic images were acquired in tapping mode using silicon probes (PPP-LFMR, Nanosensors) with a normal spring constant of 0.2 N·m^−1^ and a tip radius of ≈8 nm. Raman spectra were collected using a 533 nm laser (0.5 mW power) to avoid sample damage. The surface roughness (Ra) values of MoS_2_ were measured from the corresponding topographic images in an area of 1 μm × 1 μm.

### Friction measurements

The atomic-scale stick–slip frictional behaviors were characterized by measuring the lateral force versus the scanning distance over 3–5 nm (to capture atomic-scale features), a friction loop composed of a complete trace and retrace scan over the same line was obtained. The applied loads of the tip for the atomic-scale stick–slip measurement were set to 10–100 nN, and the velocities were set to 10–40 nm·s^−1^.

To ensure data reliability, (1) AFM tip wear was excluded by measuring tip–MoS_2_ adhesion before and after friction test (adhesion remained constant ±5% for all tests), (2) normal and lateral forces were calibrated via the non-contact method [20] to quantify absolute force values, (3) all tests were performed under ambient conditions (20–30 °C, 30–40% relative humidity) to avoid environmental interference, and (4) all friction measurements were performed on relatively flat regions of the MoS_2_ films, and the AFM tip was calibrated to ensure a constant tip radius during all measurements.

To quantify the sub-scale slip distance, the slip phase of nanoscale stick–slip motion was first extracted. Then a linear baseline was fitted for the scanning distance axis of the slip phase to eliminate the slight drift of the AFM scanning system. The slip distance was defined as the projected length of the slip phase segment on the calibrated scanning distance axis (from the start point to the end point of the slip phase). This spatial displacement-based quantification method is tailored to the unique characteristics of atomically thin MoS_2_, unlike rigid materials (e.g., NaCl(001)) where discrete lateral force jumps enable force drop-derived slip distance calculation [21]. A force drop-based approach would introduce non-negligible errors due to the absence of separable force jump peaks. Our method prioritizes direct spatial displacement measurement to avoid force calibration uncertainties, ensuring sub-nanoscale accuracy for the slip distances targeted herein. This approach aligns with the physical definition of sub-nanoscale slip distance as the actual tip displacement between adjacent sub-sites, which is critical for characterizing load- and layer thickness-dependent sub-nanoscale stick–slip motion.

## Results and Discussion

**Figure 1:**
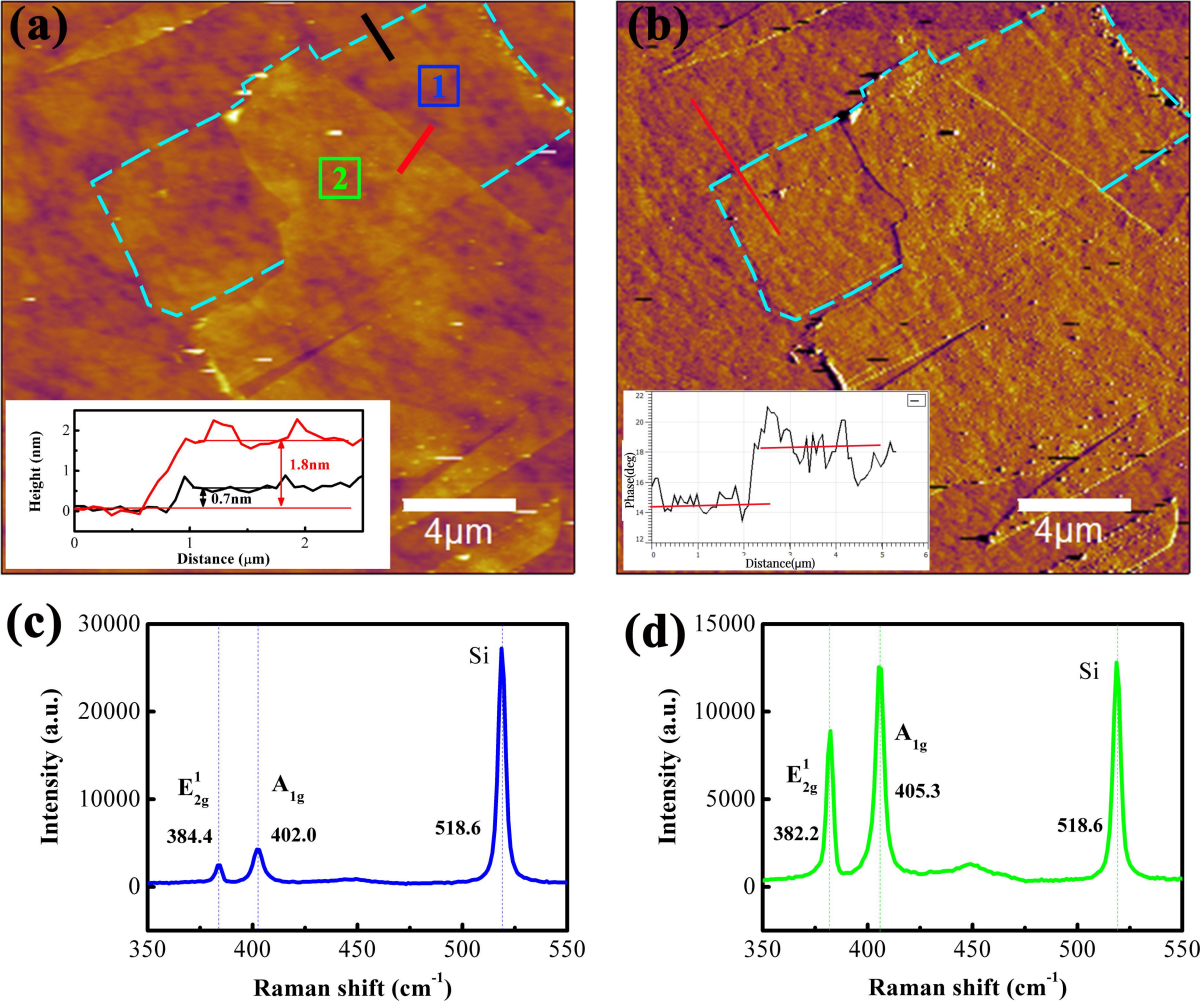
(a) AFM topographic image of atomically thin MoS_2_ along with the cross-sectional height profiles in the insets. (b) AFM phase image corresponding to the topographic image along with the cross-sectional profiles in the insets. (c, d) Raman spectra measured in the areas marked with “1” and “2”, respectively.

Due to the relatively large fluctuation of atomically thin MoS_2_ caused by the rough substrate, the thickness of MoS_2_ on the rough substrate is determined by combining the cross-sectional height profile and the Raman spectra. The height profiles in topographic image are carefully located in the fluctuation direction of rough substrate. Thus, the thicknesses of MoS_2_ on the rough substrate along the black and red height profiles are about 0.7 and 1.8 nm, respectively. Figure 1a shows an experimentally obtained typical AFM topographic image of atomically thin MoS_2_ on the SiO_2_/Si substrate, with cross-sectional height profiles measured from the same sample in the insets. The rough substrate (Ra = 0.605 ± 0.079 nm) complicates direct thickness identification of 1L MoS_2_; thus, phase imaging (Figure 1b) was used to distinguish MoS_2_ from the substrate (based on the contrast from differences in tip–sample interaction). Cross-sectional profiles (Figure 1a inset) show two distinct thicknesses of 0.7 and 1.8 nm. The theoretical thickness of 1L MoS_2_ is 0.62 nm [22]. However, considering the uncertainty of the AFM measurement, the measured thickness of single-layer MoS_2_ may be larger than the theoretical value [22–23]. Therefore, it can be roughly concluded that the MoS_2_ area with the thicknesses of 0.7 nm may correspond to one layer, and the other measuring area corresponds to four layers.

Raman spectra were measured to further determine the number of MoS_2_ layers on substrate. Two main characteristic peaks of MoS_2_ are clearly presented, which correspond to the 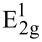 and A_1g_ modes. The Raman shifts of A_1g_ and 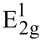 peaks changes slightly with the number of MoS_2_ layers. The characteristic peak of SiO_2_/Si substrate is also present at 518.6 cm^−1^. The difference of the Raman shift between A_1g_ and 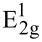 peaks is about 19 cm^−1^ (Figure 1c), which indicates that the MoS_2_ area with the thickness of 0.7 nm is 1L [24]. The difference of about 23 cm^−1^ (Figure 1d) indicates the corresponding MoS_2_ area is composed of 3–4 layers. Combined with topographic data, the two regions in Figure 1a were identified as 1L and 4L, respectively.

Surface roughness (Ra) measurements (within an area of 1 μm × 1 μm) showed that both 1L and 4L MoS_2_ reduce the substrate roughness: Ra = 0.518 ± 0.056 nm (1L), 0.288 ± 0.081 nm (4L), and 0.605 ± 0.079 nm (substrate). The higher roughness of 1L MoS_2_ is attributed to the influence of the substrate, which induces wrinkle deformation, a factor that modulates tip–MoS_2_ contact geometry and friction. Substrate roughness induces slight strain and wrinkle deformation of MoS_2_ films, which slightly expands the stick–slip period. However, this modulation is a systematic effect for both 1L and 4L MoS_2_ under all load conditions, and it does not change the relative variation trend of the strengthening effect and sub-nanoscale stick–slip motion with load and layer thickness.

**Figure 2:**
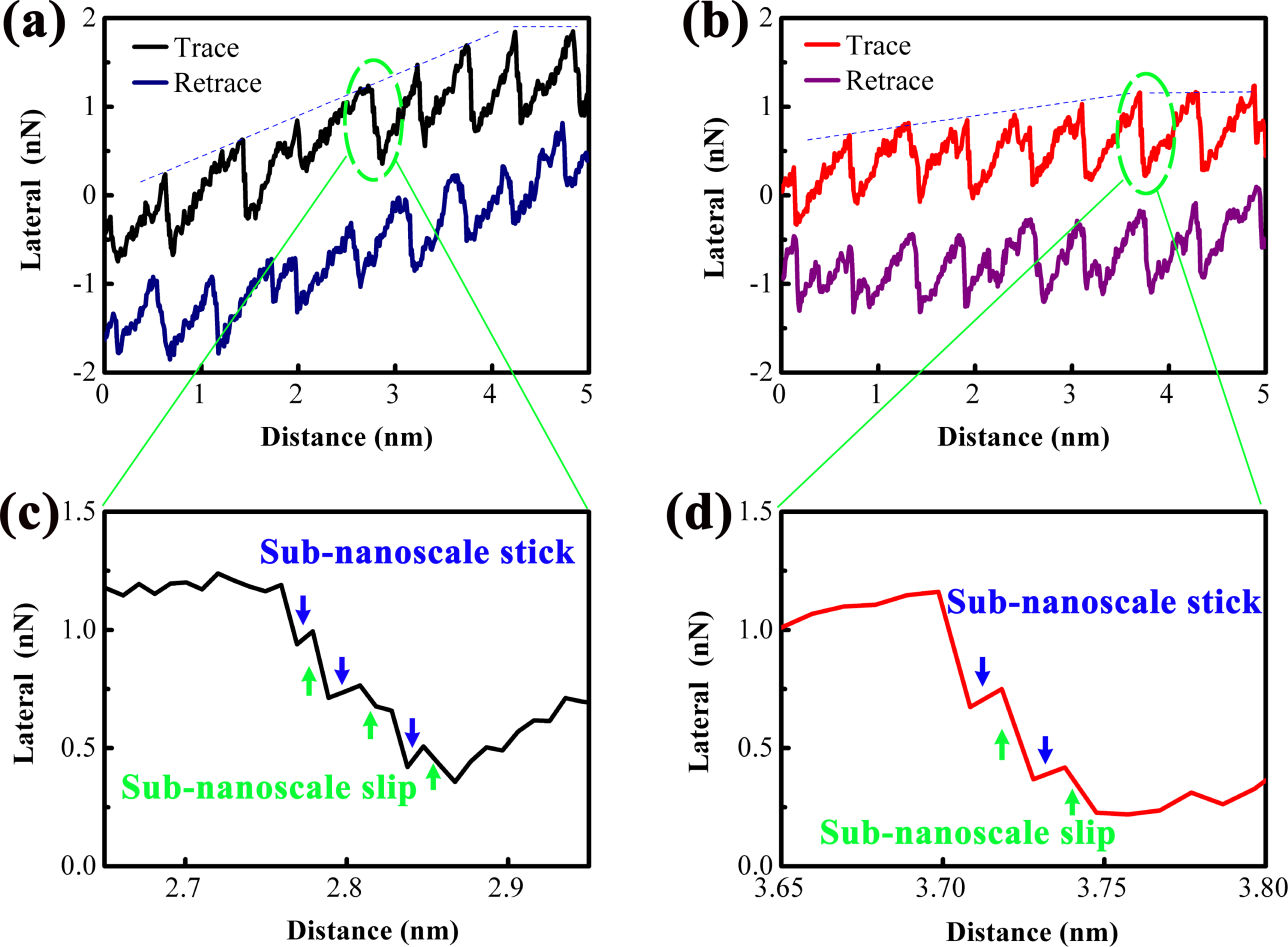
Lateral forces versus scanning distances showing the atomic-scale stick–slip behavior measured on (a) 1L MoS_2_ and (b) 4L MoS_2_. (c, d) Expanded images of the local nanoscale slip motion corresponding to the green ovals in (a) and (b), respectively. The slopes of the dashed trending lines in (a) and (b) represent the degree of strengthening effect. The blue and green arrows in (c) and (d) indicate the sub-nanoscale stick and slip motions, respectively.

Figure 2a and Figure 2b show lateral force vs scanning distance curves for 1L and 4L MoS_2_, respectively, under ambient load. Both curves exhibit transient nanoscale strengthening, evident from the increasing slope of the lateral force (dashed lines), which quantifies the “degree of strengthening”. The degree of strengthening is higher for 1L MoS_2_ than for 4L MoS_2_, consistent with previous observations [18–19]. This is because more layers of MoS_2_ reduce out-of-plane flexibility, limiting the ability of MoS_2_ to deform and adjust to the tip–sample contact [17,24]. For atomically thin MoS_2_ films (≤5 layers), the out-of-plane flexibility is dominated by interlayer van der Waals interactions, rather than the number of interlayer spacing regions. As the number of layers increases, the interlayer van der Waals forces increase, which rigidifies the MoS_2_ film and reduces its out-of-plane deformability. Although more interlayer spacing regions exist in thicker films, the enhanced interlayer coupling restricts the independent deformation of each layer, leading to an overall reduction in the ability of the film to conform to the AFM tip and adjust to the tip–sample contact geometry. This conclusion is consistent with the experimental observations of the strengthening effect in this work and previous studies. Notably, the stick–slip period observed in 1L and 4L MoS_2_ is slightly larger than the bulk MoS_2_ lattice period (0.31 nm) [25]. This expansion is attributed to strain induced by the rough substrate, which distorts the MoS_2_ lattice and increases the tip’s slip distance between stable sites.

A key finding is the observation of sub-nanoscale stick–slip motion during the slip phase (Figure 2c,d). In classical models, slip is instantaneous, because the tip atom sticks at an energetically stable position, and then suddenly slips to next energetically stable position when the external force exceeds the interfacial interaction [26]. However, our data show intermediate “sub-nanoscale sticks” (blue arrows) and “sub-nanoscale slips” (green arrows) within the nanoscale slip phase. It is consistent with the observation in MD simulations [11–12], where the slip motion is a complex procedure rather than a simple jumping motion. These sub-nanoscale events cause the lateral force to decrease gradually (not abruptly) during slip, increasing the total slip distance. For 1L MoS_2_, the slip distance is larger than that of 4L MoS_2_, confirming that slip distance is a valid metric for sub-nanoscale stick–slip activity.

Conventional atomistic MD simulations (with simulation box sizes typically below 10 nm) cannot capture long-range distortions (e.g., wrinkles with lengths above 100 nm) of MoS_2_ films induced by substrate adhesion, sliding velocity, and chemical composition of the tip [11–12]. These long-range distortions are present in our experimental system and modulate the tip–MoS_2_ contact geometry, which is not fully considered in the MD simulations [11–12]. Despite the above limitation, the MD simulations and our experiments reach a consistent core conclusion: Nanoscale slip motion is not an instantaneous jump but a complex process composed of multiple sub-nanoscale stick–slip events. The disagreement in the scale of distortions does not alter the fundamental mechanistic understanding of sub-nanoscale stick–slip motion.

The strengthening effect is a prevalent phenomenon observed in atomically thin 2D materials [18–19]. Its underlying mechanism is generally attributed to either the puckering effect or the evolution of interfacial contact quality, two competing yet complementary processes. Atomically thin 2D materials possess ultrahigh out-of-plane flexibility, enabling them to undergo dynamic deformation and configuration adjustment to conform to the AFM tip during sliding. Notably, this flexibility diminishes with increasing layer thickness, which gives rise to the layer-dependent nature of the strengthening effect. Thus, the transient strengthening effect can be regarded as a dynamic adaptation process of the tip–sample contact interface prior to attaining a stable sliding state. Regarding sub-nanoscale stick–slip motion, previous studies have suggested correlations with the in-plane motion of the AFM tip [11], as well as the interfacial contact area and contact geometry [12]. However, the intrinsic mechanism governing this sub-nanoscale friction behavior remains poorly understood, largely due to the lack of direct experimental observations and quantitative characterizations.

**Figure 3:**
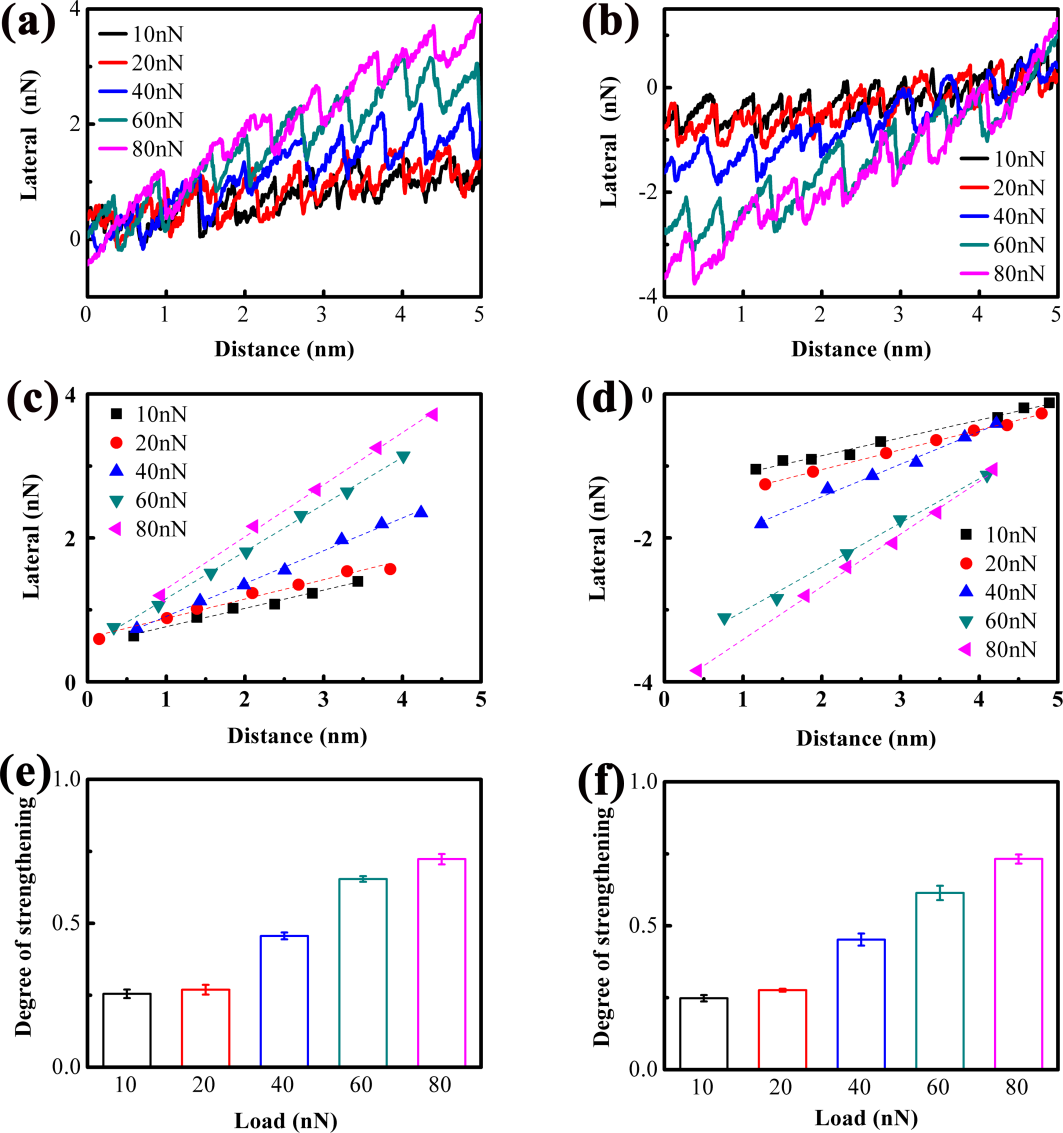
Lateral force (a) trace and (b) retrace curves measured on 1L MoS_2_ under different loads. (c, d) Peak values of lateral force in each stick–slip period extracted from the corresponding lateral force trace and retrace curves at the stage of strengthening, along with the linearly fitted lines. (e, f) Degrees of strengthening calculated from the slopes of the dashed lines.

To further visualize sub-nanoscale stick–slip motion in atomically thin MoS_2_ and unravel its underlying mechanism, systematic friction measurements were conducted under varying applied loads to investigate both nanoscale and sub-nanoscale stick–slip behaviors. Figure 3a, and Figure 3b present, respectively, the lateral force trace and retrace curves for 1L MoS_2_ across different load conditions. All curves exhibit strengthening, with the lateral force magnitude increasing with load. From the inclination of the lateral force trace and retrace curves, the strengthening effect is qualitatively inferred to intensify with increasing load. For quantitative characterization of this load dependence, the lateral force peak values within each stick–slip cycle during the strengthening stage were extracted. Figure 3c and Figure 3d depict these extracted peak values corresponding, respectively, to the trace and retrace curves in Figure 3a and 3b, respectively. The dashed lines represent linear fit results, where the slope of each line quantifies the degree of strengthening. The load-dependent degree of strengthening is summarized in Figure 3e and Figure 3f. It remains nearly constant at loads below 20 nN, followed by a linear increase with further load elevation.

**Figure 4:**
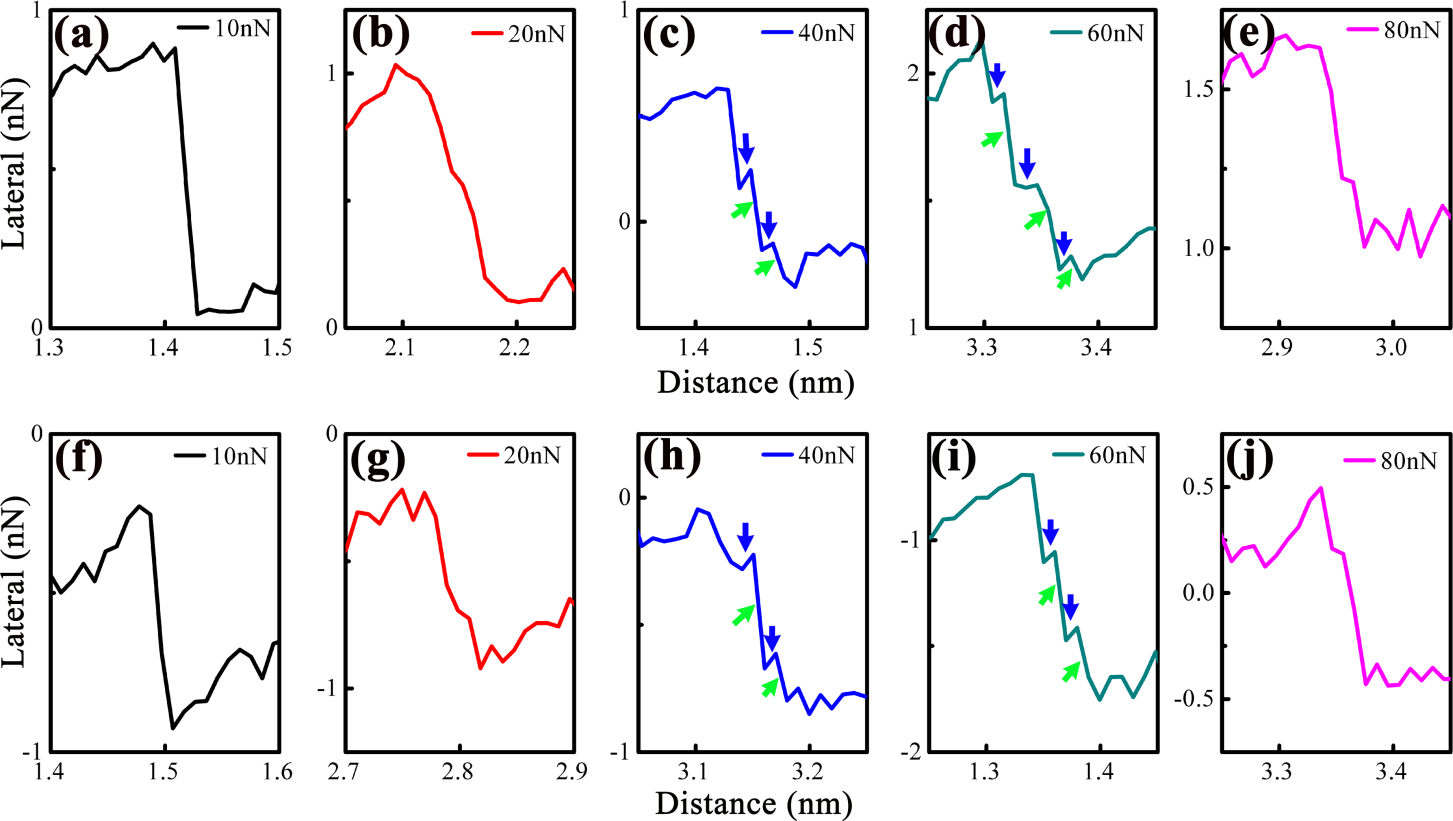
Expanded images of the chosen slip motions extracted from (a–e) lateral force trace curves and (f–j) retrace curves in Figure 3, showing the different sub-nanoscale stick and slip motions. The blue and green arrows mark out the sub-nanoscale stick and slip motions, respectively.

Figure 4a–j shows magnified slip phases (from Figure 3) to resolve sub-nanoscale stick–slip motion under different loads. At relatively low loads (10–20 nN), the slip phase is smooth (no sub-nanoscale events), indicating that tip motion is dominated by instantaneous atomic-scale slip. At moderate loads (40–60 nN), sub-nanoscale stick–slip events are prominent (green arrows), increasing slip distance. At ultrahigh load (80 nN), sub-nanoscale events vanish, and the slip phase becomes smooth again.

**Figure 5:**
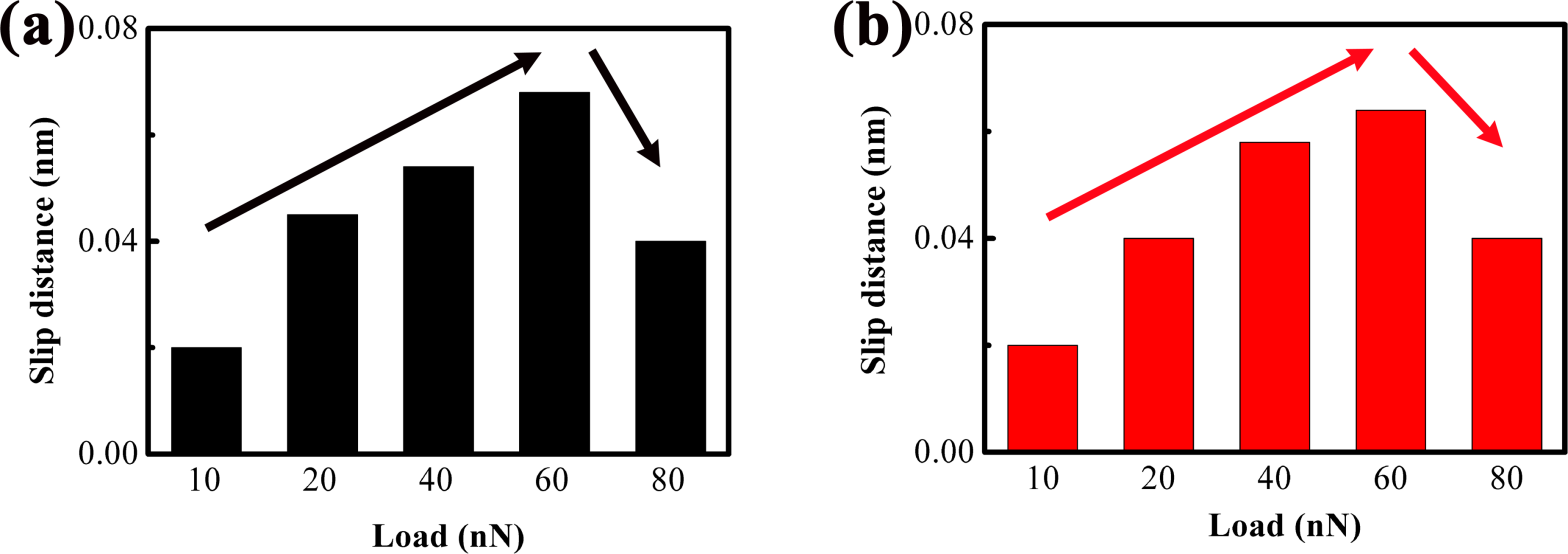
Slip distance versus load. The slip distances in (a) and (b) are obtained from the expanded lateral force trace and retrace curves in Figure 4a–e and 4f–j, respectively.

As discussed earlier, sub-nanoscale stick–slip motion contributes to an increased slip distance, as the slip line transitions from a vertical to an oblique profile during the stick–slip cycle. Figure 5 quantifies slip distance vs load for trace (Figure 5a) and retrace (Figure 5b) scans, derived from the analysis of Figure 4. It is evident that the slip distance first increases with rising load, followed by a decrease when the load exceeds 60 nN. Notably, the slip distances extracted from the trace and retrace curves exhibit a consistent trend. The initial increase in slip distance stems from the gradual enhancement of sub-nanoscale stick–slip events within the slip phase. In contrast, the subsequent decrease in slip distance is attributed to a corresponding reduction in the occurrence of these sub-nanoscale stick–slip events at higher loads. The slip distance is much smaller than the stick–slip period (bulk lattice period of MoS_2_ is 0.31 nm [25]) because it only represents the displacement within a single slip process (from one stable sub-site to another) in the sub-nanoscale, rather than the total displacement between two major atomic lattice sites.

**Figure 6:**
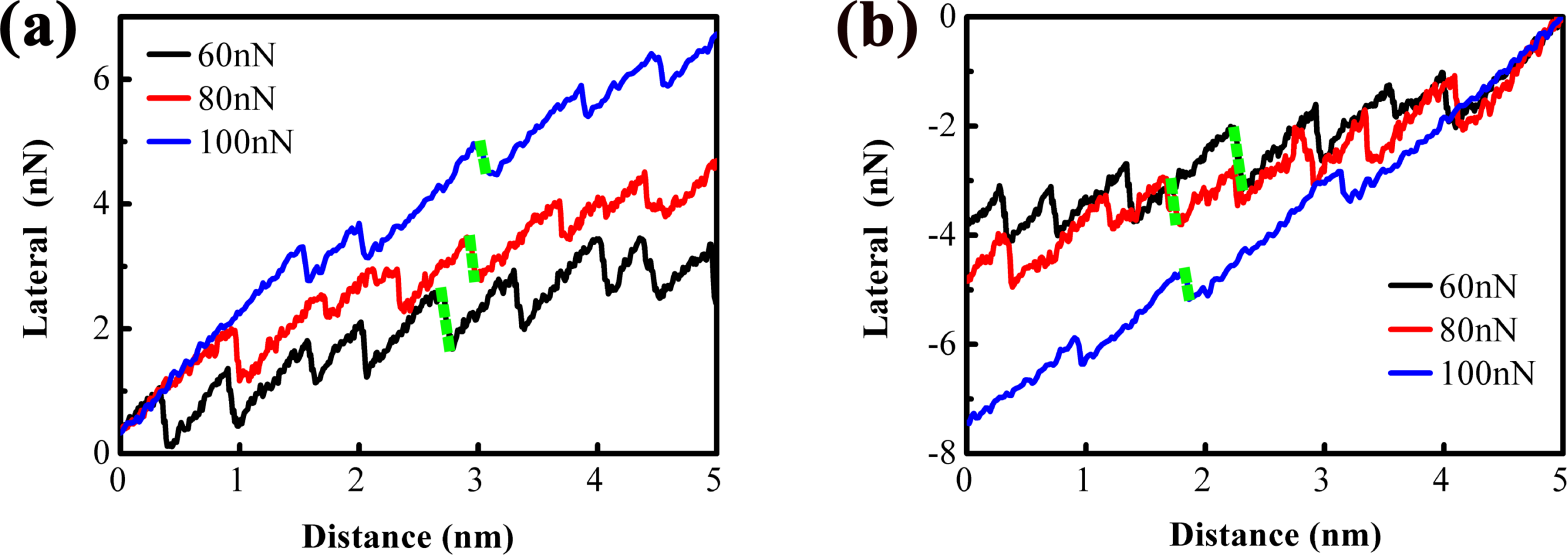
Lateral force (a) trace and (b) retrace curves measured on 1L MoS_2_ under the loads from 60 to 100 nN. The green dotted lines mark a typical nanoscale slip line, in which the length of the line represents the changing degree of lateral force in the slip phase.

The reduced occurrence of sub-nanoscale stick–slip motion at 80 nN may be associated with a diminished variation in lateral force during the slip phase. To explicitly compare the lateral force variation in the slip phase across different loads, the lateral force trace and retrace curves recorded under loads ranging from 60 to 100 nN are presented in Figure 6. Consistent with the aforementioned findings, the degree of strengthening intensifies with increasing load. Furthermore, as highlighted by the green dotted lines, the length of the slip line decreases with escalating load, an observation that indicates a reduction in the magnitude of lateral force change during the slip phase as load increases. Meanwhile, the length of the initial stick line expands with rising load, implying a load-dependent increase in static friction. It can be inferred that, when the load exceeds a critical threshold, nanoscale slip motion within the 5 nm scanning region will cease, with static friction replacing the nanoscale stick–slip motion. Consequently, the attenuation of nanoscale slip motion under relatively high loads leads to a corresponding decrease in slip distance.

**Figure 7:**
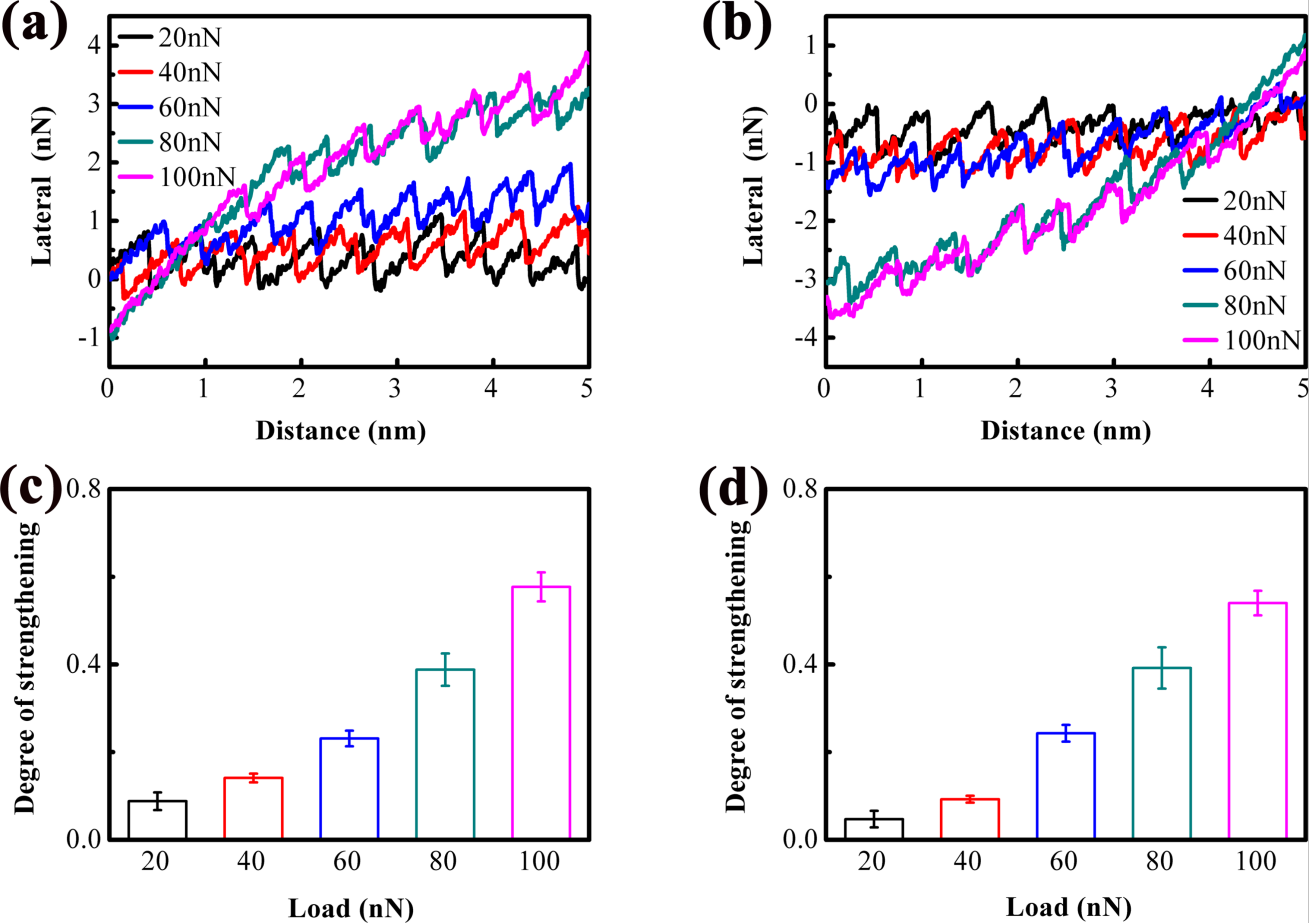
Lateral force (a) trace and (b) retrace curves measured on 4L MoS_2_ under different loads. (c, d) Degrees of strengthening calculated from the corresponding lateral force curves.

Given that the contact area between the AFM tip and atomically thin MoS_2_ is proportional to the applied load [3] and that it also depends on the number of MoS_2_ layers [18], 4-layer (4L) MoS_2_ on a rough substrate was employed to further investigate nanoscale and sub-nanoscale friction behaviors. Figure 7a and Figure 7b display, respectively, the lateral force trace and retrace curves for 4L MoS_2_ under various load conditions. The strengthening effect is nearly negligible at loads below 20 nN, so only the lateral force curves recorded at loads above 20 nN are presented herein. Notably, as the load increases from 20 nN, the strengthening effect emerges and progressively intensifies with further load elevation. Figure 7c and Figure 7d summarize the corresponding degree of strengthening, calculated using the same method as described earlier. It is evident that the degree of strengthening is relatively weak at 20 nN, followed by a continuous increase with increasing load. Additionally, the variation in lateral force during the slip phase diminishes as the load rises. The degree of strengthening of 4L MoS_2_ is moderately smaller than that of 1L MoS_2_.

**Figure 8:**
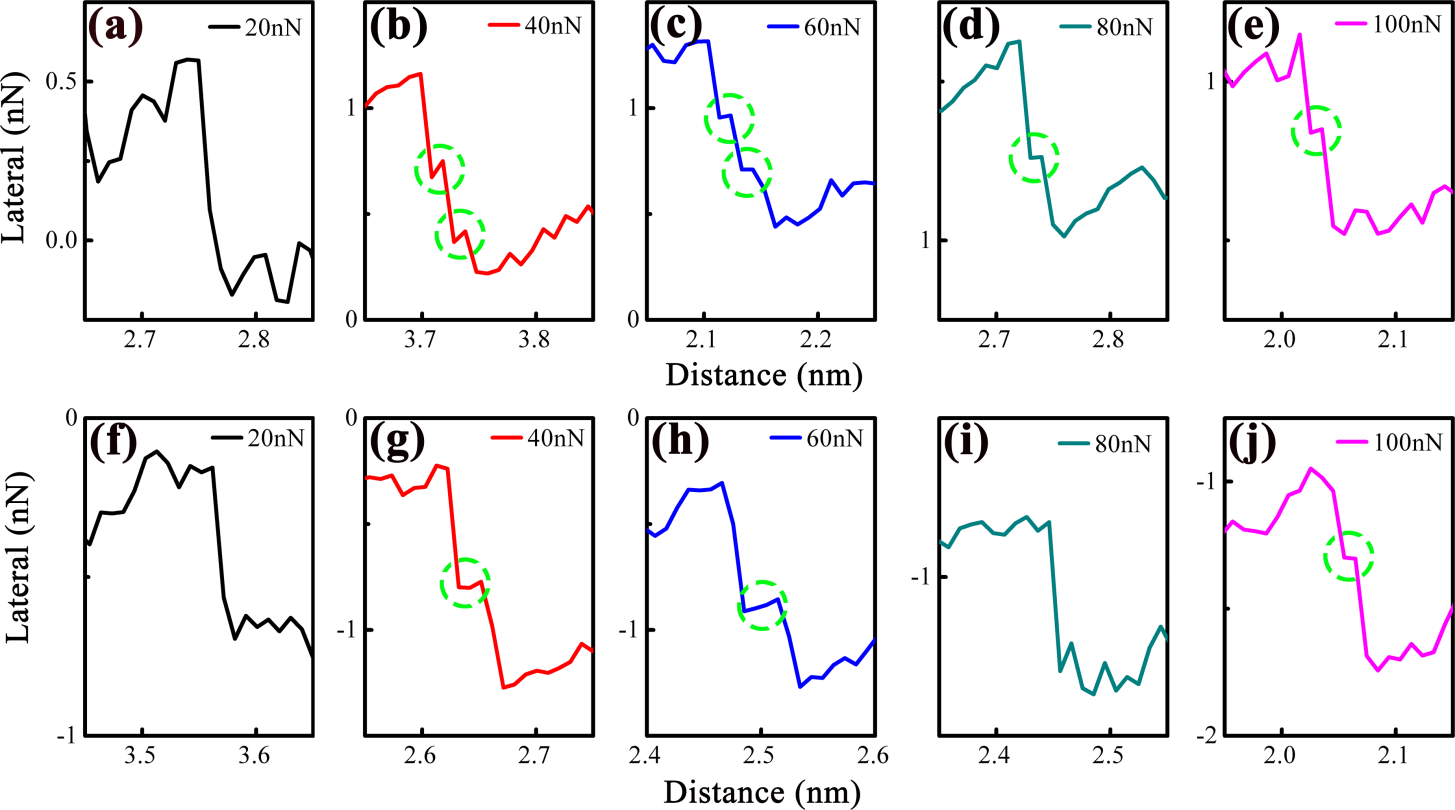
Expanded images of chosen slip motions extracted from (a–e) lateral force trace curves in Figure 7a and (f–j) retrace curves in Figure 7b, showing the different sub-nanoscale stick–slip motions. The green dotted ovals mark out the sub-nanoscale stick and slip motions.

Sub-nanoscale stick–slip motion was also identified in 4L MoS_2_. Figure 8 presents magnified views of selected slip events extracted from the lateral force trace and retrace curves in Figure 7. Notably, sub-nanoscale stick–slip motion is absent at a load of 20 nN, becomes distinctly prominent when the load increases to 40 and 60 nN, and diminishes again with further load elevation. The load-dependent variation of sub-nanoscale stick–slip motion in 4L MoS_2_ closely resembles that observed in 1L MoS_2_, suggesting a shared underlying mechanism governing this sub-nanoscale friction behavior across different MoS_2_ layer thicknesses.

**Figure 9:**
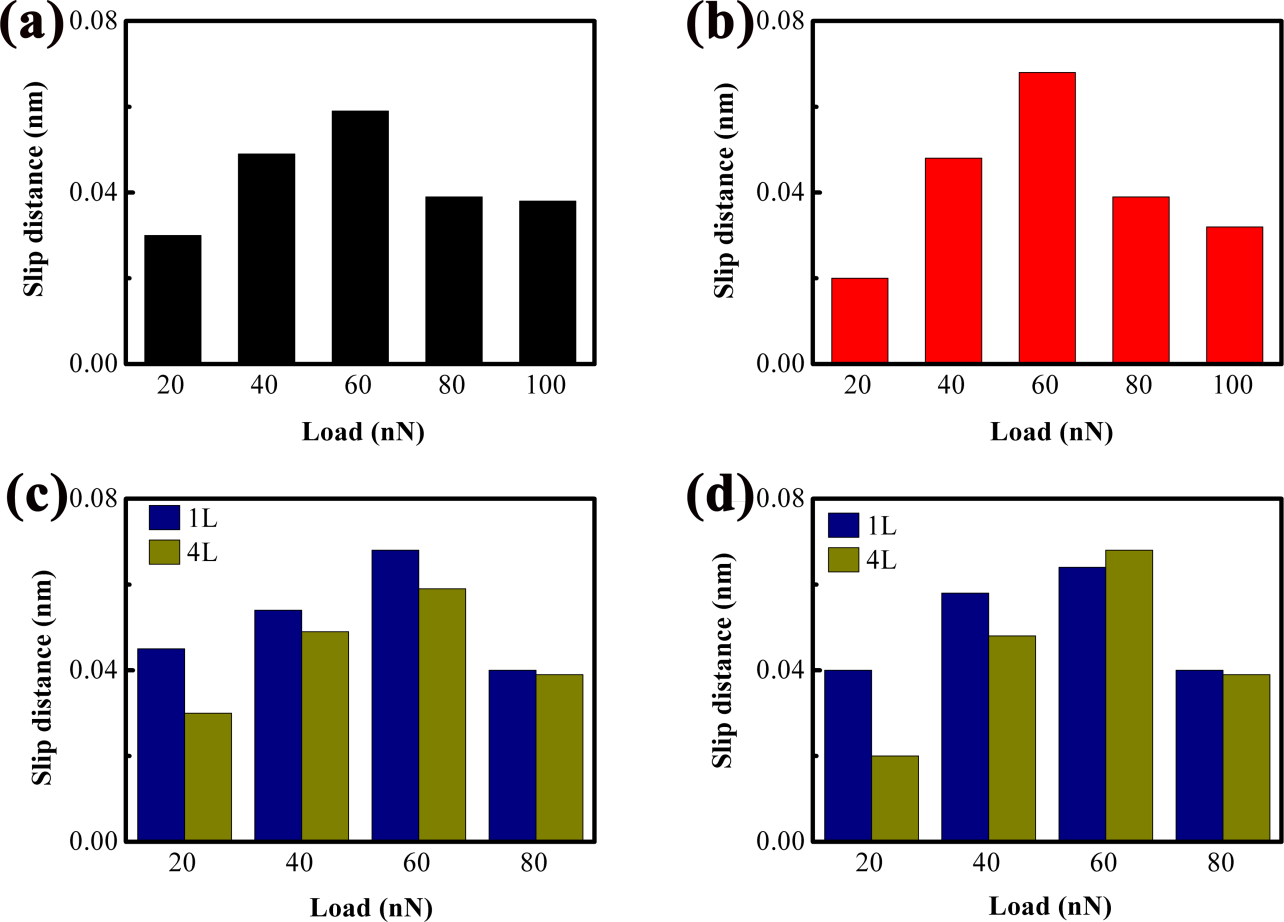
Slip distance versus load. The slip distances in (a) and (b) are obtained from the expanded lateral force trace and retrace curves in Figure 8a–e and Figure 8f–j, respectively. (c, d) Comparisons of slip distances between 1L and 4L MoS_2_ based on the lateral force trace curves and retrace curves, respectively.

The slip distances derived from the lateral force trace and retrace curves in Figure 8 are summarized in Figure 9a and Figure 9b, respectively. Consistent with the trend observed for 1L MoS_2_, the slip distance of 4L MoS_2_ first increases with rising load, followed by a decrease once the load exceeds a critical value. This initial increase in slip distance is attributed to the gradual enhancement of sub-nanoscale stick–slip events within the slip phase. Additionally, Figure 9c and Figure 9d present a direct comparison of slip distances between 1L and 4L MoS_2_. It is evident that the slip distance of 4L MoS_2_ is moderately smaller than that of 1L MoS_2_ under the same load conditions, an observation that aligns with the layer-dependent differences in friction and strengthening effect discussed earlier.

For the 4L MoS_2_ film, the bottom layer is strongly adsorbed on the SiO_2_/Si substrate via van der Waals interaction, while the upper three layers are less strongly bound to each other. During AFM sliding, the tip drags the top layer, and the weak interlayer van der Waals force can induce interlayer stick–slip motion between adjacent layers (each layer slides with a gradually decreasing velocity from the tip to the substrate). These interlayer stick–slip events are an important source of the sub-nanoscale stick–slip motion observed in the 4L MoS_2_ system. However, for the 1L MoS_2_ system, there is no interlayer structure, so the sub-nanoscale stick–slip motion is solely attributed to the in-plane motion of the tip apex and the modulation of tip–sample contact geometry [11–12]. This is the fundamental reason why the slip distance of 4L MoS_2_ is smaller than that of 1L MoS_2_ under the same load: The interlayer stick–slip events in 4L MoS_2_ are restricted by the interlayer van der Waals force, leading to a smaller cumulative displacement.

Mechanisms underlying the nanoscale and sub-nanoscale stick–slip friction behaviors have been systematically discussed. For the nanoscale strengthening effect in atomically thin MoS_2_, two primary mechanisms are widely recognized, namely, the puckering effect [18] and the evolution of interfacial contact quality [19]. However, the distinct roles and boundaries of these two mechanisms remain incompletely clarified.

Notably, the puckering effect rooted in load-dependent contact area modulation [3,26–27] is predominantly governed by the applied load, while the evolution of interfacial contact quality is primarily associated with sliding velocity [28]. This distinction is reflected in the load dependence of the strengthening effect. Under relatively low loads, the strengthening effect is nearly load-insensitive, indicating that the evolution of interfacial contact quality dominates this regime. Specifically, atomically thin MoS_2_ exhibits the capacity to dynamically adjust its atomic configuration during initial sliding, thereby enhancing tip–sample pinning and commensurate contact quality. This dynamic adaptation gradually strengthens interfacial interactions, ultimately giving rise to the transient strengthening effect. Concurrently, the atomic configuration adjustability of MoS_2_ diminishes with increasing layer thickness, which accounts for the layer-dependent nature of the strengthening effect under low loads.

In contrast, when the load exceeds a critical threshold, the strengthening effect intensifies monotonically with further load elevation, signaling that the puckering effect becomes the dominant mechanism. As the AFM tip slides, out-of-plane puckering deformation gradually forms in front of the tip. This deformation expands with increasing load, leading to a larger interfacial contact area and a more pronounced strengthening effect. Additionally, the relatively rough substrate employed in this study can further amplify the puckering effect under high loads. Similarly, the extent of puckering deformation decreases with increasing MoS_2_ layer thickness, which explains the layer-dependent strengthening behavior observed under high-load conditions.

Analyzing the origin of sub-nanoscale stick–slip motion in atomically thin MoS_2_ poses considerable challenges, primarily due to the ultrasmall time and length scales involved. According to molecular dynamics simulations by Yoon et al. [11], this sub-nanoscale motion is strongly correlated with the in-plane displacement of the AFM tip apex, a motion that can be modulated by the applied load, as inferred from the experimental trends observed herein. Furthermore, follow-up simulation studies [12] have revealed a strong dependence of sub-nanoscale stick–slip motion on interfacial contact area and contact geometry. These simulations demonstrated that the energy state of the AFM tip is governed by interactions with the second-closest MoS_2_ layer rather than the immediately adjacent (closest) layer, and this interlayer coupling is responsible for triggering sub-nanoscale stick–slip events. Consistent with these simulation insights, the load-dependent sub-nanoscale stick–slip motion observed in our experiments can be attributed to load-induced changes in both contact area and contact geometry. As the applied load increases, the contact area between the AFM tip and MoS_2_ expands accordingly, while the interfacial contact geometry undergoes concurrent modifications. Specifically, the interaction strength between the tip and the second-closest MoS_2_ layer is enhanced with increasing load. Additionally, the relatively rough substrate employed in this study may further amplify these interlayer interactions, thereby promoting the occurrence of sub-nanoscale stick–slip motion.

Regarding the attenuation of sub-nanoscale stick–slip motion at ultrahigh loads, this phenomenon is likely associated with the transition from nanoscale stick–slip motion to static friction. As static friction becomes dominant, the variation in lateral force during the slip phase is significantly suppressed, leading to a corresponding reduction in slip distance and the quenching of sub-nanoscale stick–slip events.

It is worth noting that the MFP-3D system used in this work has a maximum sampling rate of 1 MHz (time resolution of 1 μs), which is indeed much lower than the picosecond-scale sub-nanoscale stick–slip motion predicted by MD simulations [11–12]. This temporal resolution limitation means that our experiment cannot capture the transient dynamic process of individual sub-nanoscale stick–slip events, but only the cumulative spatial and force response of these events during the slip phase of nanoscale stick–slip motion. Notably, the slip distance (a spatial metric) we quantified in this work reflects the total displacement generated by the superposition of multiple picosecond-scale sub-nanoscale stick–slip events within the microsecond-scale sampling window of the AFM. Although the temporal details of individual sub-nanoscale events are not resolvable, the load-dependent variation trend of slip distance still accurately characterizes the evolution of sub-nanoscale stick–slip activity (i.e., the frequency and intensity of sub-nanoscale events). This spatial characterization is sufficient to reveal the core mechanism of load and layer thickness modulation on sub-nanoscale stick–slip motion, which is the key scientific focus of this work.

## Conclusion

This work reports the experimental quantification of sub-nanoscale stick–slip motion in atomically thin MoS_2_ and establishes its load- and layer thickness-dependent mechanisms using calibrated AFM. A nanoscale strengthening effect intensifies with increasing load and weakens with increasing MoS_2_ layer thickness. Under low loads (<20 nN), the evolution of interfacial contact quality (dynamic atomic configuration adjustment) dominates; under high loads (>20 nN), the puckering effect (out-of-plane deformation) becomes dominant.

Sub-nanoscale stick–slip motion was resolved experimentally in atomically thin MoS_2_, with slip distance as a reliable metric. The slip distance is constant at low loads (no sub-nanoscale events), increases at moderate loads (enhanced sub-nanoscale events via puckering), and decreases at ultrahigh loads (transition to static friction). Thicker MoS_2_ (4L) reduces both the degree of strengthening and slip distance, due to reduced out-of-plane flexibility, smaller contact area, and suppressed puckering.

These findings advance the understanding of 2D material tribology from the nanoscale to the sub-nanoscale, providing a framework for predicting and controlling friction in atomically thin materials. The load-layer thickness phase diagram for MoS_2_ friction behaviors offers critical guidance for the development of low-friction coatings, high-efficiency lubricants, and durable MEMS/NEMS.

## Data Availability

All data that supports the findings of this study is available in the published article and/or the supporting information of this article.
